# Real sample temperature: a critical issue in the experiments of nuclear resonant vibrational spectroscopy on biological samples

**DOI:** 10.1107/S0909049512001380

**Published:** 2012-02-16

**Authors:** Hongxin Wang, Yoshitaka Yoda, Saeed Kamali, Zhao-Hui Zhou, Stephen P. Cramer

**Affiliations:** a Department of Chemistry, University of California, 1 Shields Avenue, Davis, CA 95616, USA; b Lawrence Berkeley National Laboratory, 1 Cyclotron Road, Berkeley, CA 94720, USA; c JASRI, SPring-8, 1-1-1 Kouto, Sayo-cho, Sayo-gun, Hyogo 679-5198, Japan; d College of Chemistry and Chemical Engineering, Xiamen University, Xiamen 361005, People’s Republic of China

**Keywords:** nuclear resonant vibrational spectroscopy, real sample temperature(s), cryogenic adhesive, heat transfer, X-ray radiation damage

## Abstract

There are several practical and intertangled issues which make the experiments of nuclear resonant vibrational spectroscopy (NRVS) on biological samples difficult to perform. The sample temperature is one of the most important issues. In NRVS the real sample temperatures can be very different from the readings on the temperature sensors. In this study the following have been performed: (i) citing and analyzing various existing NRVS data to assess the real sample temperatures during the NRVS measurements and to understand their trends with the samples’ loading conditions; (ii) designing several NRVS measurements with (Et_4_N)[FeCl_4_] to verify these trends; and (iii) proposing a new sample-loading procedure to achieve significantly lower real sample temperatures and to balance among the intertangled experimental issues in biological NRVS measurements.

## Abstract 2

The real sample temperatures during the nuclear resonant vibrational spectroscopy on biological samples have been assessed and significantly reduced (116 → 52 K) by improving the sample-loading procedures.

## 1. Introduction

Nuclear resonant vibrational spectroscopy (NRVS) is a relatively new X-ray spectroscopy. It scans an extremely monochromatic (∼1 meV) X-ray beam through the ^57^Fe nuclear resonance at 14.4 keV, and measures the corresponding creation (Stokes) or annihilation (anti-Stokes) of phonons (Yoda *et al.*, 2001 ▶; Sturhahn, 2000 ▶; Smith *et al.*, 2005 ▶; Xiao *et al.*, 2005 ▶; Guo *et al.*, 2008 ▶; Cramer *et al.*, 2006 ▶; Scheidt *et al.*, 2005 ▶). It has several distinguished advantages in comparison with other traditional vibrational spectroscopic techniques such as infrared (IR) and Raman spectroscopies (Smith *et al.*, 2005 ▶; Xiao *et al.*, 2005 ▶; Guo *et al.*, 2008 ▶; Cramer *et al.*, 2006 ▶). It provides an element- and isotope-specific probe to the interested sites. Furthermore, it has an almost perfect selection rule to probe only the vibrational modes involving the motion of the nuclear absorber, which is ^57^Fe in this case. Normal mode analysis can simulate both frequencies and intensities in a spectrum, providing better credence to the simulated force constants. In practical aspects, it is water-transparent in comparison with far-IR spectroscopy, and it is free of fluorescence problems in comparison with resonance Raman spectroscopy. All these properties make NRVS an excellent pin-point tool for studying iron-specific chemistry and biochemistry in complicated biological molecules (Cramer *et al.*, 2006 ▶).

**Figure 1:**
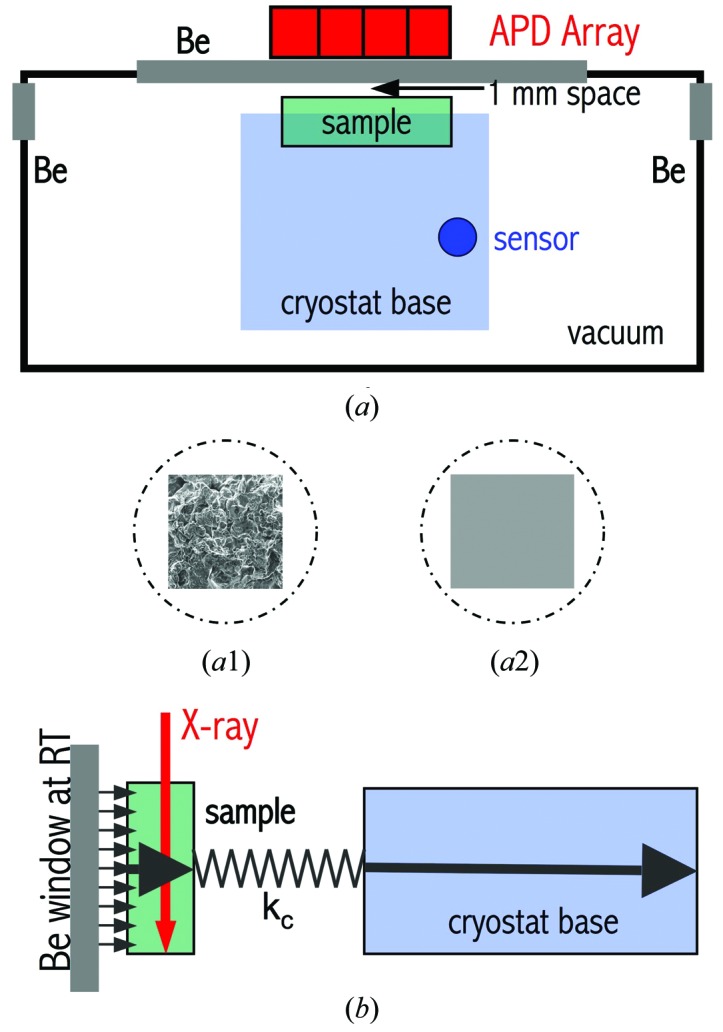
From top to bottom: (*a*) a typical NRVS experimental set-up at BL09XU at SPring-8. The inserts (*a*1 and *a*2) are imaginary pictures for a point-to-point (*a*1) and for a surface-to-surface (*a*2) contact between the sample and the cryostat base (see text for details). (*b*) A simplified heat-transfer model for a typical cold-finger cryostat.

Biological samples (or sensitive chemical samples in general) have several practical issues which often make their NRVS experiments difficult. A higher sample temperature will lead to faster radiation damage and less stable nascent chemical states (*e.g.* photochemically or electrochemically produced states), making the sample temperature the leading issue in almost every biological X-ray experiment. However, it is not a simple matter to lower the sample temperature in a NRVS experiment measuring biological samples. Issues of the NRVS signal levels, the samples’ air sensitivity and the samples’ real temperatures are all intertangled. These issues are described in detail as follows:

(i) Most biological molecules have very low metal concentration and hence a very weak spectral signal. For a NRVS experiment, this issue is even worse because the extremely monochromatic beam has an intensity of the order of 10^9^ photons s^−1^, at least three orders weaker than radiation intensities used in other synchrotron techniques. The cross section for scattering spectroscopy is often very small, making the signal even weaker. Thus it is essential for a biological NRVS experiment to increase the probing solid angle as much as possible, which requires the distance in between the sample and the cryostat window (*d*) to be as short as possible, *e.g.* ∼1 mm (see Fig. 1*a* ▶). Even with such an extreme configuration, it takes about 12–48 h to collect a biological NRVS spectrum. The use of a cold-finger cryostat and an extremely short distance in between the sample and the cryostat window (at room temperature) could lead to an elevated sample temperature during a NRVS measurement.

(ii) Many biological molecules are air-sensitive. Protein samples are often prepared and quenched in liquid nitrogen (LN_2_), 77 K, and are kept at this temperature. During the sample loading to the cryostat base, the sample would be exposed to air at a temperature higher than 77 K. To minimize this potential problem, experimentalists need to (*a*) shorten the sample-loading time, defined as the period between the samples leaving the LN_2_ and the samples being attached firmly to the base (and the cryostat being cooled down); (*b*) maintain the cryostat base at a temperature as low as possible.

In a cold-finger-type cryostat there are two current practices for mounting biological NRVS samples onto the cryostat base. The first method is to use cryogenic adhesive to attach the sample onto the base. In this method there will be no extra materials in front of the NRVS samples, so the sample–detector distance can be as short as possible. Also, the time to mount samples is very short (∼15 s). However, the cryostat base has to be warmed up above the melting point of the cryogenic adhesive. If the loading temperature is too low, the adhesive will become too sticky, and the sample–base contact could be a point-to-point contact rather than a surface-to-surface contact [Fig. 1(*a*1) *versus* Fig. 1(*a*2)].

The second method is to contact the samples onto the cryostat base by mechanical force (such as using two to four screws). With this method the cryostat base can be kept at an as-low-as-possible temperature, but the sample loading takes time (1–2 min on average). During this period the sample temperature is uncertain. The screws make the sample–window distance slightly longer, resulting in the signal being slightly weaker. A more sophisticated mechanical device may be able to load the sample faster, but will unavoidably extend the sample–window space and thus further lower the signal level during the NRVS measurements.

As illustrated in Fig. 1(*b*) ▶, there are two major sources of heat load onto the sample during the NRVS measurements: the X-ray beam and the room-temperature (RT) black-body radiation at the cryostat window. At equilibrium, heat load = heat flow = *k*_c_(*T*_2_ − *T*_1_), where *k*_c_ is the heat transfer coefficient and *T*_2_ and *T*_1_ are the temperatures for the sample and for the cryostat base (readings from the temperature sensor), respectively. Heat convection has a much higher *k*_c_ than heat conduction, and that is why a gas-exchange-type cryostat has a lower *T*_2_ than a cold-finger-type cryostat. However, in a biological NRVS measurement, using a gas-exchange cryostat or even just increasing *d* seems out of the question. Improving *k*_c_ under the current situation and thus lowering *T*_2_ becomes the only possibility. Besides, owing to the limited *k*_c_, there could be a significant difference between *T*_1_ and *T*_2_ in a NRVS measurement.

(iii) X-rays can cause radiation damage to sensitive samples (Garman & Nave, 2009 ▶; Holton, 2009 ▶; Meents *et al.*, 2010 ▶). The issue of radiation damage does not seem critical in NRVS at first because it uses a relatively low flux beam. Nevertheless, (*a*) having a less intense beam is not a real advantage because both the spectral statistics and the radiation damage are proportional to the total X-ray dose on the samples; (*b*) the perception that NRVS causes less radiation damage is based on the assumption that the samples in NRVS experiments have similar temperatures as the samples in other X-ray experiments. However, owing to (i) and (ii), the real sample temperature (*T*_2_) could be much higher than those read with temperature sensors (*T*_1_). Up to now, no NRVS publications have claimed their samples were really at <10 K (as EXAFS papers often did); rarely did they mention what their real sample temperatures were. Therefore, radiation damage is still a potential issue for a NRVS experiment.

Similar to the situation for probing the radiation damage, the best way to monitor the temperature of the exact X-ray-irradiated sample portion is by X-ray spectroscopy itself. In NRVS, this temperature is readily calculable using the imbalance between the anti-Stokes and the Stokes intensities: *S*(−*E*)/*S*(*E*) = exp(−*E*/*kT*). Although many other techniques can also evaluate the sample temperatures, they are not *in situ* and, more importantly, they are not under the same experimental conditions. For example, Raman spectroscopy will investigate the sample temperatures for the combination of laser and RT radiations, not for the combination of X-ray and RT radiations.

In this study, (i) various existing NRVS data (measured over the past several years) have been analyzed to assess the real sample temperatures during the NRVS measurements and to understand their trends with the samples’ loading conditions; (ii) several standard NRVS measurements with (Et_4_N)[FeCl_4_] have been performed to verify these trends; (iii) the sample-loading procedure has been changed to improve the real sample temperatures (*T*_2_). This study has illustrated how the new sample-loading procedure significantly lowers *T*_2_ and meanwhile balances all the intertangled experimental issues.

## 2. Experiments

### 2.1. NRVS measurements and analysis

^57^Fe NRVS spectra were recorded using standard procedures (Xiao *et al.*, 2005 ▶; Guo *et al.*, 2008 ▶; Cramer *et al.*, 2006 ▶) at beamline 09XU (Yoda *et al.*, 2001 ▶) at SPring-8, Japan. The monochromated photon flux at 14.4 keV was either ∼1.4 × 10^9^ photons s^−1^ with a 0.8 meV energy resolution or ∼2.5 × 10^9^ photons s^−1^ with a 1.1 meV bandwidth. The beam size was about 0.6 mm × 1 mm. NRVS data were generally measured between −30 meV and 70–100 meV (depending on the samples). Delayed nuclear fluorescence and Fe *K*_α_ fluorescence were recorded using a 2 × 2 APD detector array. The maximum resonant peak varied from sample to sample between 50 and 3000 counts s^−1^. In addition, a few NRVS spectra measured at ESRF (BL18) were also cited and discussed.

Spectral analysis was performed following the published procedure (Sturhahn, 2000 ▶; Smith *et al.*, 2005 ▶; Xiao *et al.*, 2005 ▶) using the *PHOENIX* software package (Sturhahn, 2000 ▶), where the observed raw NRVS spectra were calibrated to the nuclear resonant peak position (*E*_0_), normalized to *I*_0_, averaged and converted to the ^57^Fe partial vibrational density of states (PVDOS or DOS for abbreviation). The spectral conversion was optimized when the observed Stokes/anti-Stokes imbalance matched the imbalance calculated using the entered temperature as a parameter. Therefore, NRVS-measured temperature for the X-ray-irradiated sample portion was obtained during NRVS spectral analysis.

### 2.2. Sample-loading procedures

The cryostat base was maintained at a cryogenic temperature (4–7 K) using a liquid-helium-flow cold-finger cryostat. The base temperature was raised to a few K above the cryogenic adhesive’s melting point during the sample loading. Lucite boxed samples were directly attached onto the recessed trench on the top of the cryostat base with cryogenic adhesive (Fig. 1*a* ▶).

Our old sample-loading procedures used HIVAC-G cryogenic grease (Shin Etsu Chemical, melting point = 175 K) as adhesive, denoted LT grease hereafter. The LT grease is still widely used in the NRVS community as it is convenient to use over a wide range of temperatures (including RT). Procedure A1: all the critical or sensitive biological samples, such as CO-bound nitrogenase *etc.*, were loaded from LN_2_ onto the cryostat base, which was kept at 180 K at the time of the sample loading. This was to ensure an as-low-as-possible sample temperature during the loading processes. Procedure A2: ordinary biological samples, such as iron–sulfur proteins, were loaded from LN_2_ onto the cryostat base, which was kept at 190 K instead. Procedure A3: chemical complexes were loaded from RT onto the base kept at 200 K. Procedure A4: the first calibration sample (Et_4_N)[FeCl_4_] was loaded before cooling down the cryostat, *i.e.* both the sample and the cryostat base were at RT.

As will be discussed in detail in §3, LT grease has a fetal dilemma between the sample-loading temperature and the real sample temperature *T*_2_. To lower both temperatures, a lower-melting-point adhesive has to be used. Solvent 1-propanol with a melting point at 147 K (Wako Pure Chemical Industries) was selected as the new cryogenic adhesive. In our new sample-loading procedures, the cryostat base was kept at <150 K for all the samples. Procedure B1: all the protein samples were loaded from LN_2_ onto the cryostat base. Procedure B2: all the chemical complexes {including the calibration sample (Et_4_N)[FeCl_4_]} were loaded from RT onto the cryostat base.

### 2.3. Samples

Powder sample [Et_4_N][FeCl_4_] was synthesized following the published procedures (Smith *et al.*, 2005 ▶) and was used as the standard sample in this study. Other complexes and enzyme samples (Table S1)^1^ were either synthesized or received from collaborators. As the sample temperature is the subject of this study, the sample details are omitted here.

## 3. Results and discussions

### 3.1. NRVS spectra

**Figure 2:**
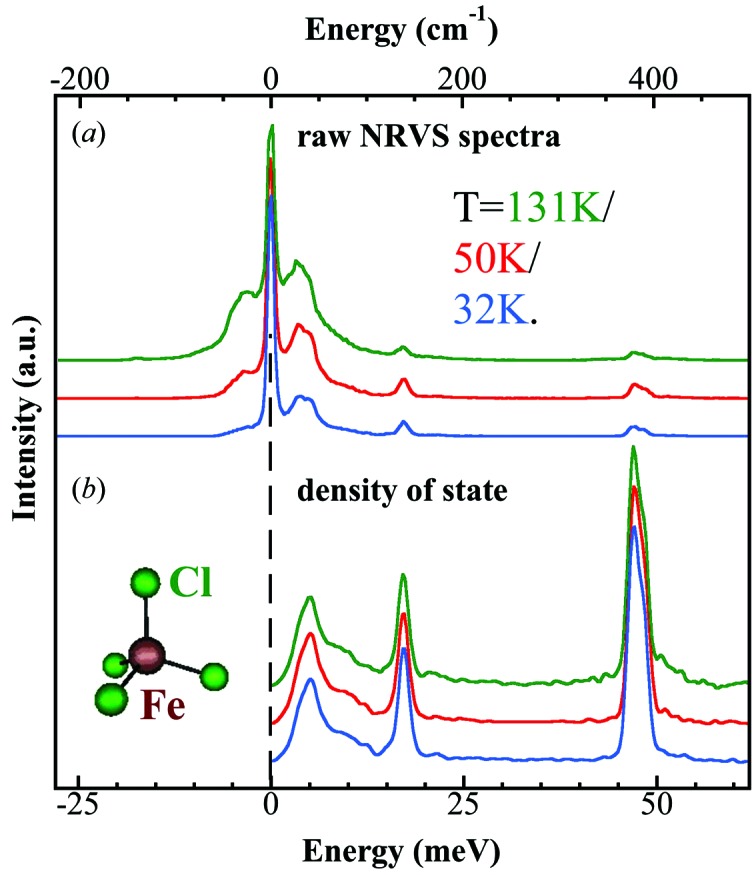
Raw NRVS spectra (*a*) and partial vibrational density of states spectra (*b*) for [FeCl_4_]^−^ at 131 K (green, top curves in each panel), 50 K (red, middle curves in each panel) and 32 K (blue, bottom curves in each panel).

The tetrahedral [FeCl_4_]^−^ ion presents the simplest vibrational spectrum for a transition metal complex. Its NRVS as well as IR and Raman spectra have been reported previously (Smith *et al.*, 2005 ▶). The [FeCl_4_]^−^ NRVS spectra and the PVDOS under different experimental conditions are illustrated in Figs. 2(*a*) (raw NRVS) and 2(*b*) ▶ (PVDOS). There are intramolecular modes near 17.1 and 47.1 meV (138 and 380 cm^−1^), a lattice mode near 5 meV (40 cm^−1^), and some residual NRVS intensity between 5 and 14 meV (40–112 cm^−1^). The highest-frequency modes have the greatest NRVS amplitude and hence the largest amount of iron motion (Smith *et al.*, 2005 ▶). As illustrated, the raw NRVS spectra are very different at different sample temperatures as the population distributions are a function of sample temperature. On the other hand, the converted PVDOS are similar because the density of states do not change as the sample temperature changes. Besides (Et_4_N)[FeCl_4_], many other NRVS spectra were cited and analyzed in this study.

### 3.2. Real sample temperatures

**Figure 3:**
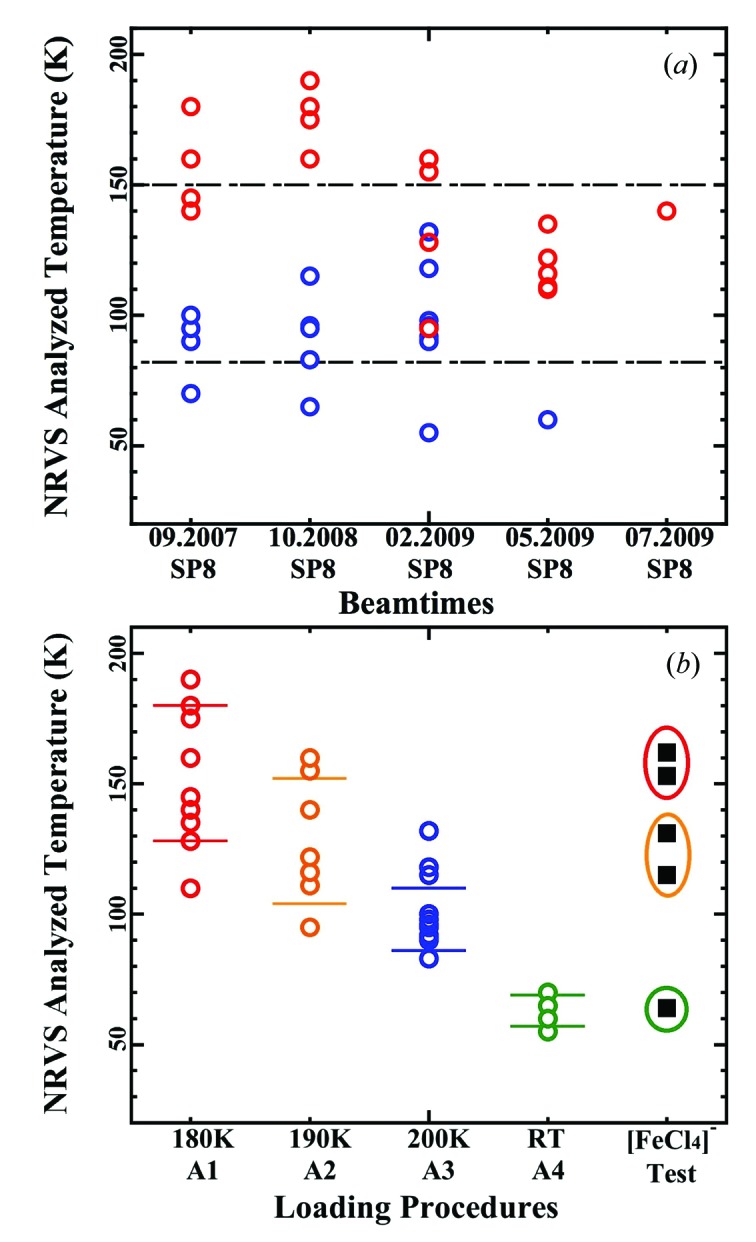
(*a*) Real sample temperatures during NRVS measurements for various samples and during various beam times using the LT grease as the cryogenic adhesive: protein samples (red circles) and chemical complexes (blue circles); the dash-dotted lines are the statistical error bar of ±1σ; (*b*) the same data as (*a*) re-illustrated against their loading procedures: A1 (180 K, red circles), A2 (190 K, orange circles), A3 (200 K, blue circles) and A4 (RT, green circles); the right-most column in (*b*) are the results from the standard NRVS tests on [FeCl_4_]^−^ (filled black squares) with loading procedures A1 (inside the red circle), A2 (inside the orange circle) and A4 (inside the green circle).

The samples’ real temperatures during the NRVS measurements, obtained by spectral analysis using *PHOENIX* (Sturhahn, 2000 ▶), are illustrated in Fig. 3(*a*) ▶. These NRVS data were collected using the old sample-loading procedures A1, A2, A3 and A4, using LT grease as the adhesive. To our surprise, the sample temperatures were in general very high. The mean temperature value was 116 K and the standard deviation for the temperature distribution (σ) was 34 K. This average does not include a few discarded data where sample temperatures >200 K were observed.

During our analysis on (Et_4_N)[FeCl_4_], we found (i) the acceptable temperature range during the process of spectral analysis is about ±5 K (1σ), and (ii) the temperature repeatability for the same sample in the same sample load but with different spectral scans is about ±3 K (1σ). The total error bar for the temperature analysis depends on various issues, and the above two values or their sum should not be treated as the total error bar but rather as a reference for the repeatability under certain experimental condition(s). Nevertheless, it cannot be concluded that the large temperature deviation of σ = 34 K is simply due to a large error bar either.

With more careful analysis, the above temperature data further illustrated the following trends: (i) model chemical complexes (blue circles), in general, had lower sample temperatures than protein samples (red circles); (ii) the first calibration measurements on (Et_4_N)[FeCl_4_] in each beam time always obtained very low sample temperatures (blue circles below the −1σ line). It has a sample temperature that is lower than other chemical complexes and even lower than the same (Et_4_N)[FeCl_4_] sample measured later during the same beam time; (iii) all the temperature data points which were above the +1σ line were from the critical protein samples, such as CO-bound nitrogenase and H_2_-bound Hmd hydrogenase *etc.*

The first possible explanation for protein samples having higher temperatures could be that the lower counts s^−1^ protein NRVS spectra could lead to an inaccurate sample temperature. For example, a few noise counts in the higher-energy region in a weak (protein) NRVS spectrum could contribute to a large error bar in calculating sample temperatures. Although it sounds reasonable, a higher error bar does not mean a higher temperature itself. Besides, this proposal could not explain (i) why all the initial calibration measurements (in each beam time) always had the lowest temperatures (lower than other chemical complexes) and (ii) why all the critical protein samples had the highest temperatures (higher than other protein samples).

The trends with the experimental conditions appear when the real sample temperatures were re-classified according to their sample-loading procedures and re-illustrated as in Fig. 3(*b*) ▶. The 180 K loaded samples (A1, red circles) have the highest mean sample temperature of 154 K with a statistics-alone error bar of ±26 K (<34 K); the 190 K loaded samples (A2, orange circles) have a sample temperature of 128 ± 20 K; the 200 K loaded samples (A3, blue circles) have 98 ± 12 K while RT loaded ones (A4, green circles) have 63 ± 6 K. This suggests that the different sample temperatures are due to the samples’ loading conditions rather than to the samples’ chemical natures (proteins *versus* complexes).

All the critical biological samples were loaded at 180 K (in an effort to save the samples). At 180 K the LT grease became very sticky and froze almost immediately after applying. Although macroscopically the sample stuck to the cryostat base, microscopically it could have a point-to-point contact rather than a surface-to-surface contact with the cryostat base [Fig. 1 ▶, (*a*1) *versus* (*a*2)], hindering the heat transfer (*k*_c_) and resulting in a higher sample temperature (*T*_2_). A large error bar (σ = ±26 K) in this loading procedure (A1) also suggests that the thermal contact situation be random.

The less-critical protein samples were loaded at 190 K and the model complexes were loaded at 200 K (both referring to the temperatures of the cryostat base). Most complex samples themselves were at RT before loading. At these loading temperatures the LT grease had less viscosity and facilitated a better surface-to-surface contact between the sample and the base. Assuming a fixed cryostat base temperature (say *T*_1_ = 7 K), a point-to-point contact will lead to a poorer thermal conduction (a lower *k*_c_) and a higher sample temperature (*T*_2_) while a surface-to-surface contact will lead to a better thermal conduction (a higher *k*_c_) and thus a lower *T*_2_. The initial calibration sample (Et_4_N)[FeCl_4_] was always loaded at RT before the cryostat was cooled down and thus had a very nice surface-to-surface contact and should have a very low *T*_2_, which is indeed the case. The decreasing statistical error bars also suggest better thermal contacts between the sample and the base when the sample-loading procedure changes: A1 → A2 → A3 → A4.

Now we can propose the following: the large statistical deviation of σ = 34 K for all the samples is not due to a large error bar or due to the differences in samples’ natures, but due to the systematical differences between different sample-loading procedures.

### 3.3. Re-evaluating the trends with (Et_4_N)[FeCl_4_]

To further prove the above speculation that the samples’ loading procedures rather than the samples themselves actually affect *T*_2_, we made several standard NRVS measurements with the (Et_4_N)[FeCl_4_] sample (∼2000 counts s^−1^), mimicking the four loading procedures (A1, A2, A3 and A4). In one case (A1), the LT grease was applied to the cryostat base at 180 K and the (Et_4_N)[FeCl_4_] sample was soaked in LN_2_ before loading onto the cryostat base. This is to simulate the real situation for loading the critical protein samples. Such mounted (Et_4_N)[FeCl_4_] indeed had very high sample temperatures (154 K and 162 K) as shown in Fig. 3(*b*) ▶ (square symbols inside the red circle); in another case (A2), the base was kept at 190 K to simulate the situation for loading regular protein samples. Such mounted (Et_4_N)[FeCl_4_] has a *T*_2_ of 131 K and 116 K (squares inside the orange circle), lower than those loaded at 180 K but still much higher than those loaded at RT (A4, the square inside the green circle).

Although other mechanisms cannot be absolutely ruled out, the adhesive-affected sample–base thermal conductivity was the major reason for the high sample temperatures in our NRVS measurements with the old sample-loading procedures (especially A1, at 180 K). A lower loading temperature led to a higher sample temperature during the NRVS measurement, and a higher loading temperature led to a lower sample temperature. Unfortunately, most biological samples need to be loaded at a temperature even lower than 180 K. Therefore, it is necessary to search for an alternative adhesive which has a freezing point lower than 175 K.

### 3.4. New sample-loading procedures

Grease-form adhesives are easy to use at all temperatures. However, no such adhesive was found to have a freezing point lower than the LT grease, *i.e.* 175 K. Then liquid organic solvents were also considered. Although the organic solvents are not adhesive at all at RT, it is an excellent bonding medium at cryogenic temperatures. In our new sample-loading procedures, 1-propanol was selected as the cryogenic adhesive because it has a low freezing point of 147 K and a relatively high boiling point of 370 K, a suitable viscosity constant and no hazards to the experimenters. Our experimental tests showed that 1-propanol became fluid at ∼140 K and the best sample-loading temperature was about 145–150 K. This made the sample-loading temperatures at least 30 K lower than the ones using the LT grease as adhesives, *i.e.* 180 K.

**Figure 4:**
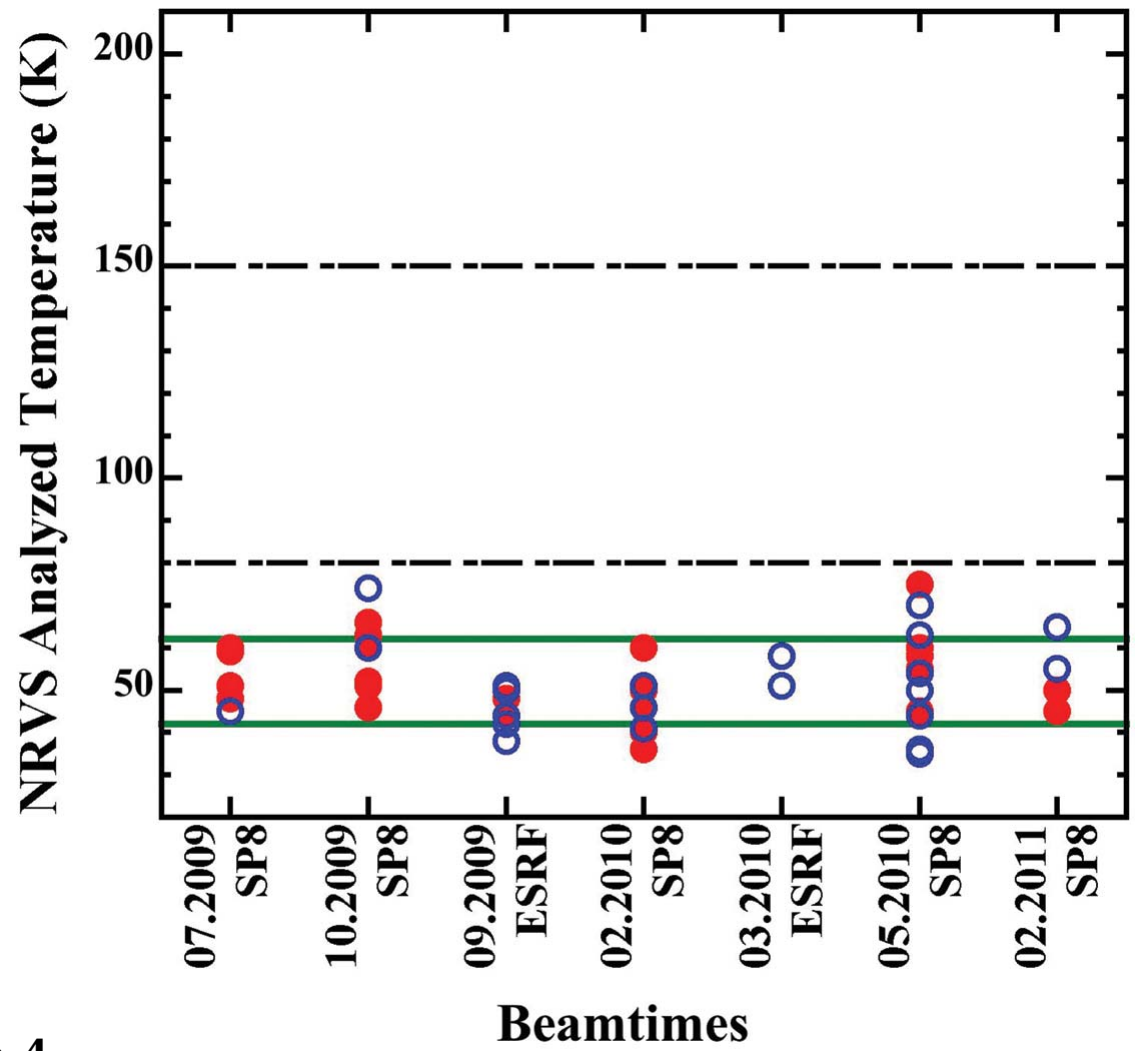
Real sample temperatures using 1-propanol as the cryogenic adhesive (proteins = filled red circles; chemical complexes = open blue circles). When the adhesive was changed from the LT grease to 1-propanol, the real sample temperatures reduced from 116 ± 34 K (between the two dash-dotted black lines) to 52 ± 10 K (between the two solid green lines); see the text for details.

The 1-propanol (and other solvents in general) has much better fluidity, hence providing a much better surface-to-surface contact. Therefore lower sample temperatures were expected with the use of 1-propanol as cryogenic adhesive, which was indeed the case. Using the new loading procedures immediately produced a much lower sample temperature of 50 ± 7 K during the particular beam time in July 2009 alone. Afterwards, we continued to use 1-propanol as adhesive and measured various NRVS samples in several beam times. As illustrated in Fig. 4 ▶, the samples loaded with 1-propanol show an average real sample temperature of 52 ± 10 K, much lower than the ones loaded using the LT grease as adhesive (116 ± 34 K).

Although the total error bar is not obtainable in this study, the above statistical error bar of ±10 K is based on seven beam times and 51 various samples, and is repeatable. Also, using 1-propanol as the adhesive, the proteins and chemical complexes have almost the same mean sample temperature and almost the same standard deviation (52.3 ± 10.8 K *versus* 51.8 ± 9.1 K). This further concluded that the new sample-loading procedures led to a better and more stable surface-to-surface thermal contact in between the sample and the cryostat base, under which all the samples have a similar and low temperature.

The rate of the radiation damage reaction is proportional to exp[−(1/*kT*)], which is the reason why X-ray experiments should be performed at as-low-as-possible temperatures. According to Garman & Nave (2009 ▶), large movements of regular atoms would be suppressed at 100 K because the amorphous solvent at cryo-temperatures is a glass with rigidly bound atoms. Even for locally sensitive EXAFS, much less radiation damage was found at the temperatures of 7–40 K in comparison with that at 100 K (Garman & Nave (2009 ▶) (but not much difference was found between 7 and 40 K). Therefore, lowering the real sample temperature from 116 ± 34 K to 52 ± 10 K is a significant step to control the possible samples’ radiation damage.

Table 1 ▶ lists some common organic solvents, which have freezing points of <175 K. Besides 1-propanol, 2-butanol and ethanol were also tested with (Et_4_N)[FeCl_4_] during two NRVS beam times with a loading temperature of ∼160 K. Those samples were found to have about the same real sample temperatures as those loaded with 1-propanol. Lower *T*_2_ was not detected.

It must be noted that the improvement on *T*_2_ was made possible owing to the better fluidity of the solvents *versus* the LT grease at low temperatures. It is not because the solvents have better thermal conductivity than the LT grease. Thus it is not likely to lower the sample temperature further simply by choosing different solvents. The different solvents are listed and discussed here just in case a different sample-loading temperature is required. For example, dimethylether has a melting point of 134 K.

How about a comparison between the 1-propanol loaded samples and the mechanically attached samples? At APS (ID03), the NRVS samples were mounted mechanically. The advantage of this procedure is that the cryostat base can be cooled to an as-low-as-possible temperature. However, the temperatures during the NRVS measurements for the similar samples were found at 70–110 K (or ∼90 ± 20 K), lower than those loaded with the LT grease but still much higher than those loaded with 1-propanol as the adhesive. As mentioned in the *Introduction* [Sec sec1], if the loading period was rushed then the quality of the sample–base thermal contact could be compromised, and the sample temperature could be even higher.

## 4. Summary remarks

In this study we have evaluated the real sample temperatures during the NRVS experiments on various biological samples and chemical complexes. We have understood the relation between the samples’ loading conditions and their real sample temperatures. Changing the cryogenic adhesive from the LT grease to 1-propanol has reduced the samples’ loading temperatures by at least 30 K (180 K → 150 K) and meanwhile reduced the real sample temperatures by 64 K (116 K → 52 K). This improvement is real, repeatable and stable.

This approach is so far the best balance among all the intertangled issues in a biological NRVS experiment, *i.e.* the as-short-as-possible sample–detector distance, the as-low-as-possible sample-loading temperatures, the as-fast-as-possible sample-loading process, and the as-low-as-possible real sample temperatures during the NRVS measurement.

Synchrotron radiation rings, undulators and NRVS beamlines continue to improve. In the future, NRVS will have a greater role in studying iron-specific chemistry/biochemistry, and meanwhile will face a greater challenge over the issue of sample radiation damage. This study begins a journey to deal with this potential problem. According to Garman & Nave (2009 ▶), lowering the sample temperature from 116 ± 34 K (above 100 K) to 52 ± 10 K is a significant step in controlling the possible radiation damage.

## Supplementary Material

Supplementary material file. DOI: 10.1107/S0909049512001380/kt5030sup1.pdf

## Figures and Tables

**Table 1:** Some common organic solvents and their properties

Solvent	Formula	Molecular weight	Melting point (K)	Boiling point (K)	Flash point (K)
2-Butanol	C_4_H_10_O	74	158	371	299
1-Propanol	C_3_H_8_O	88	147	370	288
Ethanol	C_2_H_6_O	46	159	352	286
Methanol	CH_4_O	32	195	338	285
Triethylamine	C_6_H_15_N	101	158	362	262
Tetrahydrofuran	C_4_H_8_O	72	165	339	252
Methyl *tert*-butyl ether	C_5_H_12_O	88	164	328	245
Dimethylether	C_2_H_6_O	46	134	251	232
Diethylether	C_4_H_10_O	74	157	308	228
Pentane	C_5_H_12_	72	143	309	224

## Footnotes

1 All the NRVS sample names, their experimental conditions and their NRVS analyzed derived sample temperatures are summarized in Table S1, available from the IUCr electronic archives (Reference: KT5030). Services for accessing these data are described at the back of the journal.
