# Supplementary material for: Real sample temperature: a critical issue in the experiments of nuclear resonant vibrational spectroscopy on biological samples
Source: J Synchrotron Radiat. 2012 Feb 16;19(Pt 2):257–63. doi: 10.1107/S0909049512001380 (PMC3284345; doi:10.1107/S0909049512001380)
Supplement: Supplementary file 1 [file s-19-00257-sup1.pdf]

Supplemental Materials

Table S1 NRVS Samples, their experimental conditions, and their NRVS spectra derived sample temperatures.

Type	Sample Name	Beamtime		LHe (L/hr)	T (K) reading	T (K) analyzed	Contact Media
FeCl4	FeCl4	2007.10 SP8	1	1.4	4.2	70	LT Grease
FeCl4	FeCl4	2007.10 SP8	1	1.4	4.9	90	LT Grease
H2ase	Hmd-D2	2007.10 SP8	1	1.5	4.7	145	LT Grease
H2ase	Hmd-H2	2007.10 SP8	1	1.5	4.3	160	LT Grease
Model	DFeCO	2007.10 SP8	1	1.4	4.8	95	LT Grease
Model	HFeCO	2007.10 SP8	1	1.5	4.1	100	LT Grease
N2ase	VK1Fe	2007.10 SP8	1	1.4	4.4	140	LT Grease
N2ase	VK7Fe	2007.10 SP8	1	1.5	4.2	180	LT Grease
Fe Protein	D14-O	2008.10 SP8	2	1.5	4.4	190	LT Grease
FeCl4	FeCl4	2008.10 SP8	2	1.4	5.1	65	LT Grease
H2ase	Hmd-PH6	2008.10 SP8	2	1.4	4.8	160	LT Grease
Model	DFeN2	2008.10 SP8	2	1.4	4.8	115	LT Grease
Model	Fe-N2H4	2008.10 SP8	2	1.5	4.2	83	LT Grease
Model	Fe-N2H4:15N	2008.10 SP8	2	1.5	4.4	96	LT Grease
Model	HFeN2	2008.10 SP8	2	1.4	5.2	95	LT Grease
N2ase	N2ase12CO	2008.10 SP8	2	1.5	4.2	175	LT Grease
N2ase	N2ase13CO	2008.10 SP8	2	1.5	4.4	180	LT Grease
Fe Protein	HiPiP-oxi	2009.02 SP8	3	1.5	5.2	95	LT Grease
Fe Protein	HiPiP-red	2009.02 SP8	3	1.5	5.2	155	LT Grease
Fe Protein	mdrA	2009.02 SP8	3	1.5	5.2	160	LT Grease
FeCl4	FeCl4	2009.02 SP8	3	1.4	5.2	55	LT Grease
Model	Fe-N2H4-A-15N	2009.02 SP8	3	1.4	5.2	98	LT Grease
Model	Fe-N2H4-B	2009.02 SP8	3	1.4	5.2	90	LT Grease
Model	Fe-N2H4-B-15N	2009.02 SP8	3	1.4	5.2	91	LT Grease
Model	N2ase-control	2009.02 SP8	3	1.4	5.2	118	LT Grease
Model	DNIC-1Fe	2009.02 SP8	3	1.4	5.2	96	LT Grease
Model	DNIC-1Fe-15N	2009.02 SP8	3	1.4	5.2	132	LT Grease
Model	DNIC-2Fe	2009.02 SP8	3	1.4	5.2	91	LT Grease
Model	DNIC-2Fe-15N	2009.02 SP8	3	1.4	5.2	92	LT Grease
Model	DNIC-4Fe	2009.02 SP8	3	1.4	5.2	90	LT Grease
Model	DNIC-4Fe-15N	2009.02 SP8	3	1.4	5.2	96	LT Grease
Model	Fe-N2H4-A	2009.02 SP8	3	1.4	5.2	95	LT Grease
N2ase	N2ase12CO	2009.02 SP8	3	1.5	5.2	128	LT Grease
Fe Protein	HiPiP-red	2009.04 SP8	4	1.6	4.8	111	LT Grease
FeCl4	FeCl4	2009.04 SP8	4	1.4	4.8	60	LT Grease
FeCl4	FeCl4	2009.04 SP8	4	1.4	4.8	116	LT Grease
FeCl4	FeCl4	2009.04 SP8	4	1.4	4.8	122	LT Grease
N2ase	N2ase12CO	2009.04 SP8	4	1.6	4.8	100	LT Grease
N2ase	N2ase13CO	2009.04 SP8	4	1.6	5.6	135	LT Grease
Fe Protein	MutY: native	2009.07 SP8	5	1.4	5.4	51	1-Propanol
FeCl4	FeCl4	2009.07 SP8	5	1.4	5.1	64	LT Grease
FeCl4	FeCl4	2009.07 SP8	5	1	5.4	45	1-Propanol
H2ase	Ni-A	2009.07 SP8	5	1.6	6.1	140	LT Grease
FeCl4	Test	2009.07 SP8	5	1.4	6	162	1-Propanol
FeCl4	Test	2009.07 SP8	5	1.4	6.2	153	1-Propanol
FeCl4	Test	2009.07 SP8	5	1.4	5.4	135	1-Propanol

H2ase	Ni-A: 18O	2009.07 SP8	5	1.6	5.9	48	1-Propanol
H2ase	Ni-R	2009.07 SP8	5	1	5.4	60	1-Propanol
N2ase	N2ase12CO	2009.07 SP8	5	1	5.4	59	1-Propanol
Fe Protein	D14C-NO	2009.09 ESRF	6	0.6		63	1-Propanol
Fe Protein	D14C-NO	2009.09 ESRF	6	0.6		60	1-Propanol
Fe Protein	HiPiP-O	2009.09 ESRF	6	0.6		66	1-Propanol
Fe Protein	HiPip-R	2009.09 ESRF	6	0.6		51	1-Propanol
FeCl4	FeCl4	2009.09 ESRF	6	0.6		60	1-Propanol
FeCl4	FeCl4	2009.09 ESRF	6	0.6		60	1-Propanol
H2ase	Ni-A	2009.09 ESRF	6	0.6		46	1-Propanol
H2ase	Ni-DT	2009.09 ESRF	6	0.6		52	1-Propanol
Model	Fe4S4Cl4	2009.09 ESRF	6	0.6		74	1-Propanol
FeCl4	FeCl4	2009.10 SP8	7	1	5.2	50	1-Propanol
FeCl4	FeCl4	2009.10 SP8	7	1	4.8	50	1-Propanol
H2ase	Ni-A: 18O	2009.10 SP8	7	1	5.7	44	1-Propanol
Model	Fe4S4Cl4	2009.10 SP8	7	1	4.8	44	1-Propanol
Model	Fe4S4Cl4-54/57	2009.10 SP8	7	1	5.3	38	1-Propanol
Model	Fe4S4Cl4-redo	2009.10 SP8	7	1	5.4	42	1-Propanol
Model	FePMe3	2009.10 SP8	7	1	5.2	51	1-Propanol
N2ase	APO	2009.10 SP8	7	1	5.1	48	1-Propanol
Fe Protein	3Fe-oxi	2010.02 SP8	8	1		40	1-Propanol
Fe Protein	3Fe-red	2010.02 SP8	8	1		50	1-Propanol
Fe Protein	Rieske	2010.02 SP8	8	1		36	1-Propanol
FeCl4	FeCl4	2010.02 SP8	8	1		46	1-Propanol
FeCl4	FeCl4	2010.02 SP8	8	1		41	1-Propanol
Model	FePMe3	2010.02 SP8	8	1		51	1-Propanol
N2ase	APO	2010.02 SP8	8	1		46	1-Propanol
N2ase	N2ase12CO	2010.02 SP8	8	1		41	1-Propanol
N2ase	V70A-C2H4	2010.02 SP8	8	1		60	1-Propanol
Model	DFeN2	2010.03 ESRF	9	0.6		58	1-Propanol
Model	HFeN2	2010.03 ESRF	9	0.6		51	1-Propanol
Fe Protein	D14-oxi	2010.05 SP8	10	0.7		58	1-Propanol
Fe Protein	D14-red	2010.05 SP8	10	0.7		75	1-Propanol
Fe Protein	Ferritin	2010.05 SP8	10	0.7		55	1-Propanol
Fe Protein	ToMoCNO	2010.05 SP8	10	1		45	1-Propanol
FeCl4	FeCl4	2010.05 SP8	10	0.7		50	1-Propanol
FeCl4	FeCl4	2010.05 SP8	10	1		44	1-Propanol
H2ase	CPI	2010.05 SP8	10	0.7		60	1-Propanol
Model	Fe4S4Cl4: 54/57	2010.05 SP8	10	0.7		54	1-Propanol
Model	HPTB	2010.05 SP8	10	1		36	1-Propanol
Model	HPTB: 18O	2010.05 SP8	10	1		63	1-Propanol
Model	HPTBL	2010.05 SP8	10	0.7		70	1-Propanol
Model	TP	2010.05 SP8	10	1		35	1-Propanol
Model	TP: 18O	2010.05 SP8	10	0.7		70	1-Propanol
Model	NFeN2	2011.02 SP8	11	1	20	65	1-Propanol
Model	NFeN2	2011.02 SP8	11	1	17	55	1-Propanol
N2ase	N2aseCO	2011.02 SP8	11	1	17	50	1-Propanol
N2ase	N2aseCO	2011.02 SP8	11	1	15	45	1-Propanol
